# Antibiotic-Coated Nail in Open Tibial Fracture: A Retrospective Case Series

**DOI:** 10.3390/jfmk6040097

**Published:** 2021-11-29

**Authors:** Carlo Perisano, Tommaso Greco, Chiara Polichetti, Michele Inverso, Giulio Maccauro

**Affiliations:** 1 Department of Ageing, Neurosciences, Head-Neck and Orthopedics Sciences, Orthopedics and Traumatology, Fondazione Policlinico Universitario Agostino Gemelli IRCCS, 00168 Rome, Italy; carlo.perisano@policlinicogemelli.it (C.P.); chiarapolichetti10@gmail.com (C.P.); inversomichele7@gmail.com (M.I.); giulio.maccauro@policlinicogemelli.it (G.M.); 2 Orthopedics and Traumatology, Università Cattolica del Sacro Cuore, 00168 Rome, Italy

**Keywords:** open tibial fracture, antibiotic-coated nails, antibiotic-impregnated polymethylmethacrylate, diaphyseal infections, intramedullary nails, long-bone infection

## Abstract

Implant-associated infections still represent one of the main problems in treatment of open fracture. The role of systemic antibiotic prophylaxis is now agreed and accepted; however, recent literature seems to underline the importance of local antibiotic therapy at the fracture site, and antibiotic nails have been shown to play a role in the treatment of open fractures in terms of fracture healing and lower risk of infection. We retrospectively analyzed our results, from January 2016 to March 2020, with the use of coated nails in the treatment of open tibial fractures, evaluating the rates of infection and fracture healing as primary outcomes and the rate of reoperations, time from trauma to nailing and hospital stay as secondary outcomes. Thirty-eight patients treated with coated nail (ETN Protect^TM^, Synthes) were included in the study. Minimum follow-up was of 18 months. Thirty-four of 38 patients achieved bone union and 2 patients underwent septic non-union. In our series, no systemic toxicity or local hypersensitivity to antibiotics were recorded. From this study, use of antibiotic-coated nails appears to be a valid and safe option for treatment of open tibial fractures and prevention of implant-related infections, particularly in tibial fractures with severe soft tissue exposure and impairment.

## 1. Introduction

Implant-associated infections still represent a problem for Orthopedic and Trauma surgery. Elective orthopedic surgery usually has a low infection rate (0.7–4.2%) [1]; in Trauma surgery this rate is higher with incidence rates between 3.6 and 8.1% in patients with closed fractures and between 5 and 33% in cases of open fractures [2,3]. Risk factors for infection are traumatic mechanism, severity of soft tissue injury (up to 30% in Gustilo-Anderson grade III fracture), comorbidities such as diabetes mellitus and chronic inflammatory diseases, smoking and obesity [4,5,6,7].

Implant-associated infections impair fracture healing and often involve implant removal and prolonged antibiotic therapy, often intravenously, resulting in both socioeconomic and patient quality of life impact.

Due to specific anatomical features, such as poor soft tissue coverage, poor vascularization, high-energy traumatic mechanism, bone comminution with frequent open fractures, infections are common in tibial fractures [8]. In this bone segment, open fractures showed the highest infection rate (8.8%) compared to closed fractures, where the rate reaches 2% [9].

Intramedullary nailing is the gold standard for tibial shaft fracture, with lower infection rate compared to external fixation or internal fixation with plate and screws [10].

Currently, the prevention and treatment of implant-associated infections in open fractures involves the use of high doses of systemic antibiotics which, while reducing the absolute risk of early wound infections by 60% [11], on the other can cause many systemic side effects. Systemic antibiotics can often be ineffective for two main reasons [9,12]:

(a) bacteria can colonize the implant surface forming biofilm (glycocalyx) that protects them from the action of systemic antibiotic
(b) vascular damage does not allow systemic antibiotics to reach high local concentrations.

In recent years, new strategies have been developed for prophylactic local administration of antibiotics, e.g., polymethylmethacrylate (PMMA) bone cement [13], PMMA beads [2,14], antibiotic-impregnated collagen sponges [2], polyglycolic acid or poly-DL-lactide (PDLLA)-coated implants [9,15,16] and bone allografts impregnated with antibiotics (especially useful for filling large bone defects) [17,18,19]. These implants have the advantage of ensuring, at the same time, a rapid release of antibiotics locally with high concentration at the fracture site and a lower systemic concentration of antibiotics, reducing systemic toxicity and side effects, and from the other side allow the internal fixation of the fracture [12].

Thus, recent literature has shown how antibiotic-coated intramedullary nails may play a role in treatment of open tibial fractures and in prevention of infections associated with fixation devices allowing for a high rate of bone fracture healing and reduced risk of infection [4,20,21].

The aim of this study was to evaluate the results obtained in patients with open tibial fracture treated with a gentamicin-coated nails (ETN PROtect^TM^, DePuy Synthes) [22] in terms of bone and soft tissue healing rate, infection rate, reoperation rate and hospital stay.

## 2. Materials and Methods

### 2.1. Setting

A single center multi-surgeon retrospective study was performed between January 2016 and March 2020 in a high-level Trauma Center (Fondazione Policlinico Universitario Agostino Gemelli IRCCS, Rome, Italy).

Patients who met the following inclusion criteria were enrolled in the study: open tibial fractures treated with antibiotic-coated nail (ETN PROtect^TM^, DePuy Synthes), with at least 18 months of clinical and radiographic follow-up and who had signed informed consent to treatment.

Patients with open tibial fractures not treated with antibiotic intramedullary nail, with clinical and radiographic follow-up of less than 18 months, with pre-trauma gait deficit and with other neurological pathologies that prevented adequate adherence to the protocol were excluded (Table 1).

The results were retrospectively reviewed using hospital and patient operations charts. Medical records were reviewed to gather information including patient and trauma characteristics, surgery details and surgical outcomes. The outpatient records were reviewed for post-operative follow-up. Two authors independently analyzed radiographs and clinical data.

Patient demographics including age, sex, voluptuous habits, kind of trauma, fracture type (Arbeitsge-meinschaft fur Osteosynthesefragen/Orthopaedic Trauma Association (AO/OTA) classification) [23], Gustilo-Anderson grade (GA) [5], time to nailing (TTN), eventual primary external fixation (EF) or transkeletal traction, use of negative pressure wound therapy (NPWT) with VAC (Vacuum Assisted Closure) Therapy and implant characteristics were recorded. Open fractures were subdivided by the Gustilo-Anderson classification at the time of the initial debridement in the operating room.

Preoperative and postoperative Hb and White Blood Cells count (WBC) values were collected.

### 2.2. Surgical Technique and Post-Operative Treatment

Following standard antibiotic prophylaxis (with 1st generation cephalosporin, e.g., example cefazolin), except in cases of allergies to antibiotics or in patients on antibiotic therapy targeted for previous microbiological isolations, definitive nailing was performed on a radiolucent table in supine decubitus, ensuring a flexion of at least 90° of the knee of the limb with fracture. The anesthesia, depending on the patient’s condition, was either a general or a peripheral block. Transkeletal traction devices or external fixators were removed before preparation of the sterile operating field and after extensive washing and disinfection of the skin. Access to the joint was medial, lateral, or trans- patellar tendon depending on the surgeon’s preference, patient anatomy and skin and soft tissue condition. After determining the optimal nail entry point, with closed reduction and the aid of fluoroscopy, the fractures were reduced, and the guide wire was inserted. With the dedicated instruments, the proximal housing of the nail and the reaming of the medullary canal were prepared, then we proceeded with the nailing with ETN PROtect^TM^, stabilized with percutaneous screws proximal and distal to the fractures.

In all patients a suture was performed respecting the anatomical planes, followed by sterile dressing and elastic bandage.

The indication to weight-bearing has considered both the clinical data (presence or absence of pain, soft tissue condition) and the radiographic data, encouraging early mobilization of the adjacent joints, and partial load with crutches was granted not until at least 3/4 weeks with a total weight-bearing at 8–10 weeks.

### 2.3. Outcome Measurements

Follow-up visits and radiographs were performed at 1, 3, 6, and 12 months, barring complications.

Bone consolidation was assessed clinically (pain, walking deficit) and radiographically at 3, 6 and 12 months after intramedullary nailing, evaluating the number of cortices healed (anterior, posterior, lateral, medial) on leg radiographs in the 2 standard views (anterior-posterior and lateral) including knee and ankle.

The fracture was considered completely healed when 3 or four cortices were consolidated and partially healed when 1 or 2 cortices were consolidated [24].

Data on adverse events to antibiotics (local or systemic) and infections were collected throughout the follow-up period. The evaluation was based on a clinical-anamnestic investigation with any problems reported by the patient and evaluation of liver and kidney function.

Infections were identified and classified by dividing them between surgical site infections (soft tissues) and deep infections (osteomyelitis) [25].

### 2.4. Statistical Analysis

Continuous variables were reported using means, standard deviations, and ranges.

Categorical variables were reported as an absolute value. Data were analyzed using STATA 13.0 statistical software for Windows (StataCorp. 2013. Stata Statistical Software: Release 13. College Station, TX, USA: StataCorp LP).

## 3. Results

### 3.1. Patients and Demographic Data

In our study we included a total of 38 patients who had undergone surgery with ETN PROtect^TM^ for open tibial fracture, all with at least 18 months of follow-up (mean 24.1 ± 6.1; range 18–43) (Table 2 and Table 3). Of the 38, 29 patients were men while 9 were women, with a mean age of 44.7 ± 16.6 (range 18–78). In 34 patients the antibiotic-coated nail was used for the acute treatment of the fracture, in 4 patients this implant was used for revision surgery in case of infected nonunion. According to the AO classification, 12 were 42A fractures, 17 were 42B, 7 were 42C and 1 was 43A. According to the GA classification of open fracture, 6 were classified as type I, 15 as type II, 8 as type IIIA, 5 as type IIIB and 4 as type IIIC. A fracture of the fibula was associated in 35 out of 38 patients and in 29 cases it was polytrauma.

In our series 9 were smokers and 3 were regular drug addicted.

The average preoperative Hb was 12.4 ± 1.9 g/dL (range 7.6–15.7) and the average WBC was 11.9 ± 6.0 × 10^9^/L (range 6.0–29.2).

### 3.2. Surgical Data

As Damage Control (DC) in acute 31 patients were treated with external fixation (EF) and 1 with transkeletal traction (TT) (transcalcaneal) (Table 3). Nine patients also required acute NPWT with VAC therapy for initial soft tissue treatment; of these 8 had a GA grade III and a GA II grade.

The mean time from trauma to definitive nailing (TTN) was 16.5 ± 15.3 (range 2–54) days, from this value the 4 patients who placed the antibiotic nail after revision surgery for infected pseudarthrosis were excluded, who had a much longer TTN (41 to 375 days).

Nine patients required accessory plastic surgery treatments, including 5 for skin grafting and 4 for sural flap.

The average postoperative Hb was 10.1 ± 1.4 g/dL (range 7.3–13.5) and the average postoperative WBC was 9.7± 2.8 × 10^9/L (range 4.0–14.3).

The mean hospital stay was 28.2 ± 26.9 days (range 4–128).

### 3.3. Radiographic and Clinical Outcomes

In our series, 29 patients achieved bone union within 12 months, 5 patients after 12 months, while 4 patients experienced non-union (Table 4).

**Figure 1:**
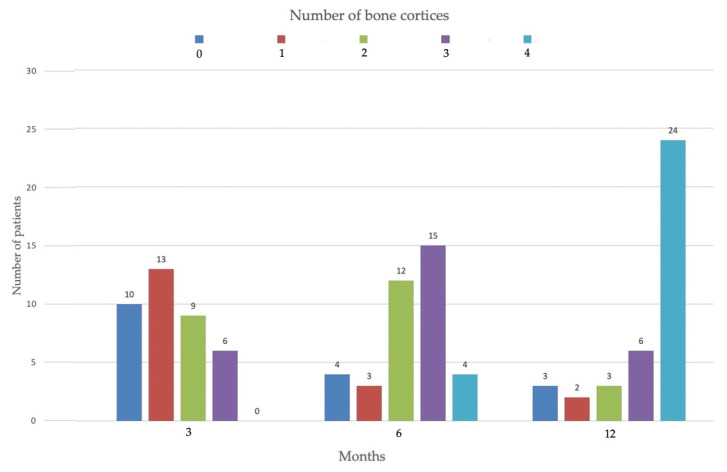
Course of bone healing. Number of bone cortices healed at 3, 6 3 12 months.

At 3 months after surgery, we found 0 bone cortices in 10 patients, 1 cortex in 13 patients, 2 cortices in 9 patients and in 6 patients there was a union of 3 cortices. After 6 months in 4 patients there were no signs of bone union (0 consolidated cortices), in 3 patients only 1 cortex was consolidated, in 12 patients 2 cortices, in 15 patients 3 cortices and in 4 patients all 4 cortices (Figure 1).

There were 13 patients with complications: the most common complication was delayed union in 5 patients, followed by 4 superficial wound and surgical site infections and 4 nonunion (2 of which were infected).

Of these 13 patients, 12 underwent further surgery, while a patient heavy smoker and drug addict suffering from non-union refused the additional proposed treatments.

The 5 patients with delayed consolidation underwent dynamization of the nail and achieved complete bone consolidation after 12 months post nailing, one of these also underwent corrective surgery for check-rein deformity [26].

Of the 4 patients with superficial infections, 3 performed a wound debridement and subsequent targeted antibiotic therapy against isolated germs, while in one case, with healing already occurred, in addition to the debridement of the wound and antibiotic therapy, the nail was removed.

Regarding the 4 patients with non-union, one refused the treatment while in the one with aseptic non-union the surgical strategy involved a new open reduction with correction of malalignment and new internal fixation with plate and screws.

The 2 patients with infected non-union underwent nail removal, debridement, bone curettage and two-stage Masquelet treatment [27].

No systemic toxicity or local hypersensitivity to antibiotics was recorded in our series.

**Figure 2:**
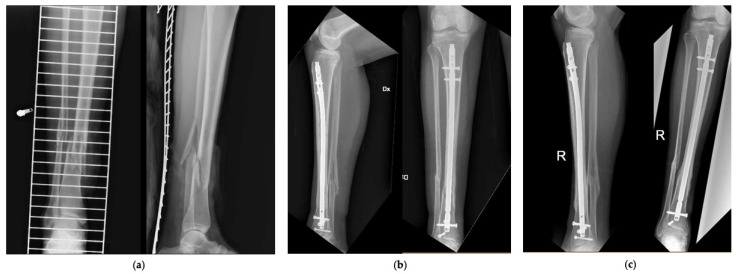
Patient number 27 of the series. (**a**) Pre-operative AP and LL *X*-rays, (**b**) AP and LL *X*-rays at 6 months, (**c**) AP and LL *X*-rays at 12 months.

**Figure 3:**
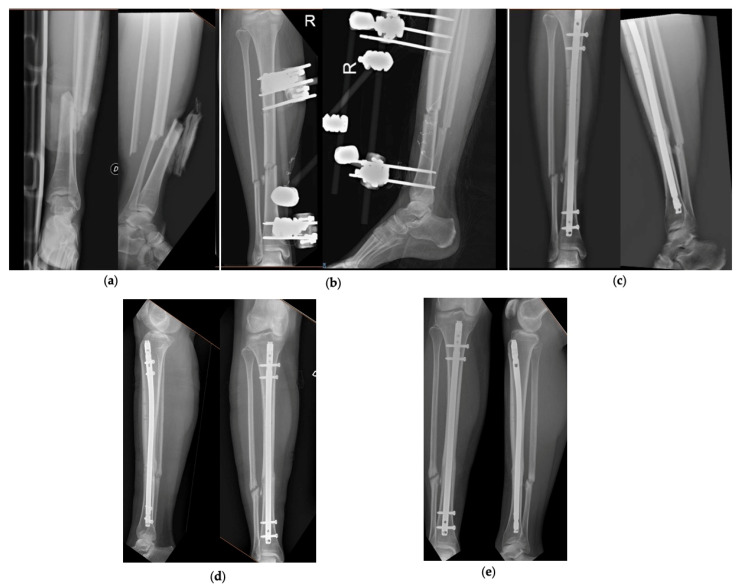
Patient number 31 of the series. (**a**) Pre-operative AP and LL *X*-rays, (**b**) AP and LL *X*-ray after application of the external fixator, (**c**) AP and LL *X*-rays at 3 months, (**d**) AP and LL *X*-rays at 6 months, (**e**) AP and LL *X*-rays at 12 months.

Figure 2 and Figure 3 show 2 clinical cases of the series, with pre-operative and follow-up radiographic images.

## 4. Discussion

Despite the innovations and good results obtained in fractures treatment, infection rate, especially in open tibial fractures, is still high and is associated with a significant socio-economic impact and a lengthening of hospitalization stay. A recent work demonstrated that infection rates and total costs for in-hospital treatment could be potentially reduced by 75% and up to 15% respectively, by using an antibiotic-coated nail in patients with high risk of infection [21,28].

Meta-analysis made by Craig had underlined the role and effects of local antibiotics showing that patients with open shaft tibia fracture who received locally delivered antibiotics as prophylaxis, in addition to systemic antibiotics, had lower infection rates than those receiving standard systemic antibiotics [4]. Literature is uniformly in agreement on the role played by the Gustilo-Anderson (GA) grade of fracture exposure on infection genesis: for the most severe case (GAIII B&C) the incidence of infections fell from over 31% with systemic antibiotics only to under 9% with the addition of local antibiotics [4].

As in literature, in our study is evident that most of patients involved in high energy trauma with open fracture are male (29 M vs. 9 F) aged between third and fifth decade. Therefore, infections related to open fractures involve more frequently males. It’s known that smoking and the others voluptuous habits, as well as diabetes mellitus, are risk fac-tors for genesis and persistence of infections because cause microvascular damage that reduces blood supply and possibility of fracture healing [29]. We didn’t find this influence in our study because only 9 patients were smokers, 3 drugs addicted, and 0 diabetes mellitus affected, so we couldn’t evaluate this association.

While acute treatment for DC in patients with polytrauma and open fractures is now standardized, with sterile irrigation, wound debridement, administration of systemic antibiotic prophylaxis and temporary stabilization of the fracture within 6–8 h of arrival in the Emergency Room, more the timing for definitive fracture treatment is controversial and less uniform in the literature. In our study, the mean time from trauma to final nailing (TTN) was about 16 days, although at first glance appears slightly long, seems justified by the complexity of the patients included, mostly polytraumatized and with need for ancillary medical and surgical care (hemodynamic stabilization, thoracic and/or abdominal surgery) which often resulted in a delay in orthopedic treatment.

The average hospital stay in our series is high, 28 days, and with a wide range (from 4 to 128 days); this would seem to make our data lose value, but, it is due to the general conditions and the need in some of these patients for accessory, medical and surgical treatments, in addition to those for tibial fractures which for some of them involved a significant lengthening of hospital stay.

Based on our other case studies [9] and on the recent study by Franz et al. [21], it seems that gentamicin-coated nails at a higher cost on the market can reduce hospitalization and the rate of re-operation especially in higher risk open fractures (GAIII).

Overall, we achieved complete bone consolidation in 34 of the 38 patients, although dynamization of the nail was required in 5 of these 34 patients. Bone consolidation was not achieved only in 4 patients, in one of these cases it was achieved after revision of the fixation with plate and screws, while in the other 2 patients a two-stage Masquelet intervention was required for bone infection and subsequent infected pseudarthrosis (both patients had a grade III fracture according to GA).

Furthermore, about soft tissue complications, only 5 out of 38 patients required wound revision surgery, with deep debridement and subsequent targeted antibiotic therapy. These results agree with Walter et al. [20] study on 13 patients with open tibial fractures treated with an antibiotic intramedullary nail; in their series 11 patients achieved bone consolidation without the need for additional surgical treatment, while 2 patients required nail removal and revision surgery for device-associated infection.

In another series, outcomes obtained with treatment with uncoated tibia nail and antibiotic-coated nail were compared: 3 patients in antibiotic-coated nail group had presented superficial surgical site infection and required a second intervention for wound debridement and subsequent targeted antibiotic therapy, 6 reoperation (3 dynamizations and 3 superficial wound debridement); regarding the bone healing rate at 12 months, 18 of 23 fractures were completely healed [9].

In our case series in 4 patients the antibiotic-coated nail was used as revision surgery for previous infected nonunion of the tibia and all 4 achieved complete bone healing, although 2 had complications (in one case with delay of consolidation successfully treated with dynamization of the nail and in the other a superficial wound infection treated successfully through debridement and targeted antibiotic therapy).

Wasko and Boren [30] in their study too used antibiotic nails in 10 patients as revision surgery following previous intramedullary nailing, with 6-year follow-up of infection eradication and bone consolidation, with no further treatment needed to control. the infection.

All this seems to show excellent results with these implants, considering the complexity of these patients and the severity of the local and general conditions.

Our study, with 38 recruited patients, is one of the largest currently in literature and, furthermore, our series is very homogeneous for fracture features (all “42” according to AO Classification except one “43”). Ours is also one of the studies with the longest follow-up in the literature, ranging from 18 to 43 months.

Despite these strengths, we recognize several limitations in our study such as the retrospective nature, the lack of a control group which does not allow comparison of these implants with other types of fixation of open tibial fractures and the small number of patients while remaining one of the studies with the largest sample in the literature.

## 5. Conclusions

From this study, use of antibiotic-coated nails appears to be a valid and safe option for treatment of open tibial fractures, enabling fracture healing and lowering the infection rate of internal fixation devices and the need for revision surgery, with consequent impact on the quality of life of patients and on the socio-economic system. However, future large-scale randomized control trials are needed to obtain statistically significant results that allow standardization of the use of antibiotic-coated tibial nails.

## Figures and Tables

**Table 1:** Inclusion and Exclusion criteria.

Inclusion Criteria	Exclusion Criteria
Open tibial fractures treated with antibiotic-coated nail	Open tibial fractures not treated with antibiotic-coated nail
At least 18 months follow-up	Less than 18 months follow-up
Informed consent to treatment	Pre-trauma gait deficit
	Neurological pathologies

**Table 2:** Results.

	ETN PROtect^TM^ (38 Patients)
**Age** (Mean ± DS, range)	44.7 ± 16.6 (18–78)
**Gender**	
Male	29
Female	9
**Voluptuous habits**	
Smokers	9
Drug addicts	3
**ETN Protect^TM^ indication**	
Acute fracture	34
Revision surgery	4
**AO Classification**	
42A	12
42B	17
42C	7
43A	1
**Gustilo-Anderson Classification**	
I	6
II	15
III	17 (8 IIIA; 5 IIIB; 4 IIIC)
**Polytrauma**	
Yes	28
No	10
**Fractured Fibula**	35
**EF/TT/VAC Therapy**	31 EF; 1 TT; 8 VAC
**Plastic Surgery**	9
Skin grafts	5
Sural flaps	4
**TTN days** (Mean ± DS, range)	16.5 ± 15.3 (2–54)
**Hospital Stay days** (Mean ± DS, range)	28.2 ± 26.9 (4–128)
**Bone union**	34
Within 12 months	29
After 12 months	5
**Non-union**	4
**Total complications**	13
Delay consolidation	5
Non-union	4 (2 infected non-union)
Surgical site infection	4
**Other treatment**	12
Nail Dynamization	5
Wound debridement + targeted antibiotic therapy	3
Revision osteosynthesis	3
Nail removal and TAT	1
**Follow-up months** (Mean ± DS, range)	24.1 ± 6.1 (18–43)

EF: External Fixation; TT: Transkeletal Traction; VAC: Vacuum Assisted Closure; TTN: Time Trauma to Nailing; TAT: Targeted Antibiotic Therapy.

**Table 3:** Patients and demographic data.

N°	Age	Sex	AO Tibia	Ga	Fractured Fibula	AO Fibula	Polytrauma	Voluptuous Habits	EF/TT/VAC Therapy
1	20	M	42B3	2	Yes	4F2B	Yes	Smoking, DA	EF
2	53	M	42C3	2	Yes	4F2B	Yes	-	EF
3	24	M	42B3	1	-	-	Yes	Smoking	-
4	67	F	42B3	3A	Yes	4F2B	-	-	EF
5	68	F	42B2	3A	Yes	4F3A	-	-	EF, VAC
6	63	M	42B2	2	Yes	4F3B	Yes	-	-
7	75	M	42A1	3B	Yes	4F2B	Yes	-	EF
8 *	63	M	42A2	3A	Yes	4F2A	Yes	-	EF
9	29	M	42B3	3A	Yes	4F2B	Yes	-	EF, VAC
10	30	M	42C3	2	Yes	4F2B	Yes	-	EF
11	46	F	42A1	1	-	-	-	-	-
12	52	F	42B3	2	Yes	4F24	-	Smoking	EF
13	43	M	42A2	2	Yes	4F2B	Yes	-	EF
14	30	M	42B2	2	Yes	4F2B	Yes	-	VAC
15	57	M	42A2	3A	Yes	4F2B	Yes	-	EF, VAC
16	22	M	42A3	3C	Yes	4F2B	Yes	Smoking	EF
17	62	M	42C3	2	Yes	4F2B	Yes	-	EF
18	50	M	42A1	3C	Yes	4F2A	Yes	-	EF, VAC
19	45	F	42A2	3B	Yes	4F2A	-	-	EF, VAC
20	65	F	42A2	1	Yes	4F2A	-	-	-
21	47	M	42C3	3A	Yes	4F3B	Yes	Smoking	EF, VAC
22	57	M	42B2	2	Yes	4F2B	Yes	-	EF
23 *	46	M	42B2	3B	Yes	4F2A	Yes	-	EF
24	26	F	42B3	3B	Yes	4F2A	-	-	EF
25	28	M	42B3	3A	Yes	4F2B	Yes	-	EF
26	31	M	42C3	3A	Yes	4F2A	Yes	-	EF
27	49	F	42B3	2	Yes	4F2B	-	-	EF
28	18	M	42B3	2	-	-	Yes	-	-
29	25	M	42B3	1	Yes	4F2B	Yes	-	EF
30	53	M	42C2	1	Yes	4F2B	Yes	Smoking, DA	EF
31	36	F	42C3	3C	Yes	4F2B	-	-	EF, VAC
32 *	40	M	42B3	3B	Yes	4F2A	Yes	-	EF, VAC
33 *	52	M	43A3	3C	Yes	4F3B	Yes	Smoking	EF
34	42	M	42A2	2	Yes	4F2A	Yes	Smoking	EF
35	78	M	42A1	2	Yes	4F2A	Yes	-	EF
36	22	M	42A3	1	Yes	4F2B	Yes	-	TT
37	58	M	42A3	2	Yes	4F2B	Yes	-	EF
38	28	M	42B3	2	Yes	4F1A	Yes	Smoking, DA	EF

AO: AO classification; GA: Gustilo-Anderson classification; EF: External Fixation; TT: transkeletal traction; Vacuum Assisted Closure; M: Male; F: Female; DA drug addict. * Patients with infected nonunion, the ETN PROtect^TM^ was used for surgical revision.

**Table 4:** Surgical and clinical outcome.

N°	TTN (days)	Nail Length × Diameter (mm)	HS	Bone Union (Number of Cortices at 3–6–12 Months)	Complications	Other Treatments	Follow-Up (Months)
1	27	360 × 11	34	Yes # (0-2-2)	Consolidation delay	Dynamization	18
2	6	375 × 12	9	Yes (1-3-3)	-	-	25
3	8	345 × 10	12	Yes (3-3-3)	-	-	18
4	7	330 × 10	12	Yes (3-4-4)	-	-	19
5	54	315 × 11	91	Yes (2-2-3)	-	-	18
6	2	375 × 11	10	Yes (2-3-3)	-	-	26
7	10	315 × 10	26	Yes (2-3-4)	-	-	21
8*	104	330 × 11	55	Yes # (1-2-2)	Consolidation delay	Dynamization + check-rein deformity correction	24
9	52	345 × 11	128	Yes (2-3-4)	-		22
10	6	340 × 9	12	No (0-0-0)	Non-union, coronal malalignment	Revision osteosynthesis (plate and screws)	43
11	12	285 × 9	59	Yes (3-3-4)	-	-	27
12	11	330 × 11	12	Yes (1-3-4)	-	-	25
13	20	375 × 12	26	Yes (2-2-4)	-	-	26
14	9	315 × 10	11	Yes (1-3-4)	-	-	26
15	9	360 × 10	54	Yes (1-2-4)	-	-	28
16	16	345 × 10	31	No (0-0-0)	Infected non-union	Nail removal, fistulectomy, debridement, curettage, Masquelet	29
17	9	330 × 9	12	Yes (0-1-4)	Superficial surgical site infection	Wound debridement + targeted antibiotic therapy	29
18	48	340 × 11	77	Yes (0-2-4)	-	-	34
19	47	285 × 9	56	Yes # (0-0-1)	Consolidation delay	Dynamization	34
20	2	315 × 10	7	Yes (1-3-4)	-	-	36
21	48	375 × 10	57	Yes (0-2-4)	-	-	36
22	9	375 × 10	12	Yes # (1-2-2)	Consolidation delay	Dynamization	18
23*	331	360 × 11	13	Yes (1-2-4)	Superficial surgical site infection	Wound debridement + targeted antibiotic therapy	18
24	8	315 × 9	14	Yes (2-3-4)	Superficial surgical site infection	Wound debridement + targeted antibiotic therapy	18
25	7	345 × 10	7	Yes (3-3-4)	-	-	18
26	13	345 × 11	17	No (0-0-0)	Infected non-union malalignment	Nail removal, Masquelet	18
27	10	345 × 10	12	Yes (3-4-4)	-	-	19
28	5	375 × 10	6	Yes (0-2-3)	-	-	19
29	23	360 × 10	26	Yes # (1-1-2)	Consolidation delay	Dynamization	20
30	9	360 × 9	15	No (0-1-1)	Non-union	Patient refused further treatment	22
31	10	340 × 10	15	Yes (2-4-4)	-	-	24
32*	41	375 × 12	42	Yes (2-3-3)	-	-	24
33*	375	315 × 10	4	Yes (1-2-4)	-	-	24
34	8	330 × 10	10	Yes (3-3-4)	Superficial surgical site infection	Nail removal, wound debridement, and targeted antibiotic therapy	25
35	2	315 × 11	27	Yes (2-2-4)	-	-	20
36	22	360 × 10	20	Yes (1-3-4)	-	-	21
37	24	375 × 13	31	Yes (1-3-4)	-	-	19
38	10	345 × 8	13	Yes (1-3-4)	-	-	25

TTN: Time Trauma to Nailing; HS: Hospital Stay. * Patients with infected nonunion, the ETN PROtect^TM^ was used for surgical revision. # Bone consolidation in more of 12 months.

## Data Availability

The study data will be available upon request to the corresponding author (email: tommaso.greco01@icatt.it).

## References

[1] Periti P., Stringa G., Mini E. (1999). Comparative multicenter trial of teicoplanin versus cefazolin for antimicrobial prophylaxis in prosthetic joint implant surgery. Italian Study Group for Antimicrobial Prophylaxis in Orthopedic Surgery. Eur. J. Clin. Microbiol. Infect. Dis..

[2] Diefenbeck M., Mückley T., Hofmann G.O. (2006). Prophylaxis and treatment of implant-related infections by local application of antibiotics. Injury.

[3] Ostermann P.A., Henry S.L., Seligson D. (1994). Timing of wound closure in severe compound fractures. Orthopedics.

[4] Craig J., Fuchs T., Jenks M., Fleetwood K., Franz D., Iff J., Raschke M. (2014). Systematic review and meta-analysis of the additional benefit of local prophylactic antibiotic therapy for infection rates in open tibia fractures treated with intramedullary nailing. Int. Orthop..

[5] Gustilo R.B., Anderson J.T. (2002). JSBS classics. Prevention of infection in the treatment of one thousand and twenty-five open fractures of long bones. Retrospective and prospective analyses. J. Bone Jt. Surg. Am..

[6] Cianni L., Bocchi M.B., Vitiello R., Greco T., De Marco D., Masci G., Maccauro G., Pitocco D., Perisano C. (2020). Arthrodesis in the Charcot foot: A systematic review. Orthop. Rev..

[7] Greco T., Polichetti C., Cannella A. (2021). Ankle hemophilic arthropathy: Literature review. Am. J. Blood Res..

[8] Metsemakers W.J., Reul M., Nijs S. (2015). The use of gentamicin-coated nails in complex open tibia fracture and revision cases: A retrospective analysis of a single centre case series and review of the literature. Injury.

[9] Greco T., Cianni L., Polichetti C., Inverso M., Maccauro G., Perisano C. (2021). Uncoated vs. Antibiotic-Coated Tibia Nail in Open Diaphyseal Tibial Fracture (42 according to AO Classification): A Single Center Experience. BioMed Res. Int..

[10] Lillo M., Ezzo O.E., Cauteruccio M., Ziranu A., Santis V.D., Maccauro G. (2019). Infections in primary intramedullary nailing of open tibial fractures: A review article. Eur. Rev. Med. Pharmacol. Sci..

[11] Gosselin R.A., Roberts I., Gillespie W.J. (2004). Antibiotics for preventing infection in open limb fractures. Cochrane Database Syst. Rev..

[12] Nizegorodcew T., Palmieri G., Marzetti E. (2011). Antibiotic-Coated Nails in Orthopedic and Trauma Surgery: State of the Art. Int. J. Immunopathol. Pharmacol..

[13] McKee M.D., Li-Bland E.A., Wild L.M., Schemitsch E.H. (2010). A prospective, randomized clinical trial comparing an antibiotic-impregnated bioabsorbable bone substitute with standard antibiotic-impregnated cement beads in the treatment of chronic osteomyelitis and infected nonunion. J. Orthop. Trauma.

[14] Schmidmaier G., Lucke M., Wildemann B., Haas N.P., Raschke M. (2006). Prophylaxis and treatment of implant-related infections by antibiotic-coated implants: A review. Injury.

[15] Fuchs T., Schmidmaier G., Raschke M.J., Stange R. (2008). Bioactive-Coated Implants in Trauma Surgery. Eur. J. Trauma Emerg. Surg..

[16] Schmidmaier G., Wildemann B., Stemberger A., Haas N.P., Raschke M. (2001). Biodegradable poly(D,L-lactide) coating of implants for continuous release of growth factors. J. Biomed. Mater. Res..

[17] Govoni M., Lamparelli E.P., Ciardulli M.C., Santoro A., Oliviero A., Palazzo I., Reverchon E., Vivarelli L., Maso A., Storni E. (2020). Demineralized bone matrix paste formulated with biomimetic PLGA microcarriers for the vancomycin hydrochloride controlled delivery: Release profile, citotoxicity and efficacy against *S. aureus*. Int. J. Pharm..

[18] Winkler H., Kaudela K., Stoiber A., Menschik F. (2006). Bone grafts impregnated with antibiotics as a tool for treating infected implants in orthopedic surgery—One stage revision results. Cell Tissue Bank.

[19] Peeters A., Putzeys G., Thorrez L. (2019). Current Insights in the Application of Bone Grafts for Local Antibiotic Delivery in Bone Reconstruction Surgery. J. Bone Jt. Infect..

[20] Walter N., Popp D., Freigang V., Nerlich M., Alt V., Rupp M. (2021). Treatment of severely open tibial fractures, non-unions, and fracture-related infections with a gentamicin-coated tibial nail—Clinical outcomes including quality of life analysis and psychological ICD-10-based symptom rating. J. Orthop. Surg. Res..

[21] Franz D., Raschke M., Giannoudis P.V., Leliveld M., Metsemakers W.J., Verhofstad M.H.J., Craig J.A., Shore J., Smith A., Muehlendyck C. (2021). Use of antibiotic coated intramedullary nails in open tibia fractures: A European medical resource use and cost-effectiveness analysis. Injury.

[22] Expert Tibial Nail (ETN)|With PROtect. https://www.jnjmedicaldevices.com/de-DE/product/expert-tibial-nail-etn.

[23] Muller M.E., Nazarian S., Koch P., Schatzker J. (1990). The AO Classification of Fractures of Long Bones.

[24] Johnson E.E., Urist M.R., Finerman G.A. (1988). Repair of segmental defects of the tibia with cancellous bone grafts augmented with human bone morphogenetic protein. A preliminary report. Clin. Orthop. Relat. Res..

[25] Horan T.C., Andrus M., Dudeck M.A. (2008). CDC/NHSN surveillance definition of health care-associated infection and criteria for specific types of infections in the acute care setting. Am. J. Infect. Control.

[26] Holcomb T.M., Temple E.W., Barp E.A., Smith H.L. (2014). Surgical Correction of Checkrein Deformity after Malunited Distal Tibia Fracture: A Case Report. J. Foot Ankle Surg..

[27] Careri S., Vitiello R., Oliva M.S., Ziranu A., Maccauro G., Perisano C. (2019). Masquelet technique and osteomyelitis: Innovations and literature review. Eur. Rev. Med. Pharmacol. Sci..

[28] Greco T., Vitiello R., Cazzato G., Cianni L., Malerba G., Maccauro G., Perisano C. (2020). Intramedullary antibiotic coated nail in tibial fracture: A systematic review. J. Biol. Regul. Homeost. Agents.

[29] Basilico M., Vitiello R., Oliva M.S., Covino M., Greco T., Cianni L., Dughiero G., Ziranu A., Perisano C., Maccauro G. (2020). Predictable risk factors for infections in proximal femur fractures. J. Biol. Regul. Homeost. Agents.

[30] Wasko M.K., Borens O. (2013). Antibiotic cement nail for the treatment of posttraumatic intramedullary infections of the tibia: Midterm results in 10 cases. Injury.

